# Loggerhead sea turtle nesting and coyote depredation habits in relation to weather and the presence of wolf urine data set

**DOI:** 10.1016/j.dib.2021.107642

**Published:** 2021-11-30

**Authors:** Michael Wauson, William Rogers

**Affiliations:** Biology Department, Winthrop University, Rock Hill, South Carolina 29733, United States

**Keywords:** Loggerhead, Coyote, Behaviour, Conservation, Tom Yawkey preserve

## Abstract

The data in this manuscript are comprised of loggerhead sea turtle nesting records and coyote depredation events on South Island Beach in the Tom Yawkey Preserve, South Carolina, from 2015 to 2019 comprise the data in this manuscript. We compared the nesting and depredation rates with abiotic factors that may have influenced them. We analysed our data using G-tests to determine whether any abiotic factors were associated with coyote depredations or the timing of loggerhead nesting. Data were collected in conjunction with an experiment testing a possible conservation tool to deter coyote depredation of loggerhead sea turtle nests (Wauson and Rogers 2021).

An Excel® file contains the entire data set in the supplemental material. Tables and their corresponding statistics test whether behavioural traits of loggerhead sea turtles and coyotes change based on certain abiotic factors, as well as how coyote depredation rates behaviour change in the presence of wolf urine.

**Specifications Table**

**Table:** 

Subject	Zoology
Specific subject area	Loggerhead nesting and coyote depredation behaviour
Type of data	Table
How data were acquired	We collected environmental and tidal data from field surveys conducted by South Carolina Department of Natural Resources Turtle Technician Teams on the Tom Yawkey Wildlife Preserve. Environmental and tidal date were collected from online databases.
Data format	Raw Analysed Filtered
Parameters for data collection	Nest type had to be identified as loggerhead sea turtle; the depredator of the nest had to be identified as coyote.
Description of data collection	A South Carolina Department of Natural Resources Turtle Technician Team collected nesting and depredation data between 2015 and 2019. We collected tidal and temperature data retroactively.
Data source location	Primary Data: Institution: South Carolina Department of Natural Resources City/Town/Region: Georgetown, South Carolina Country: United States Latitude and longitude (and GPS coordinates, if possible) for collected samples/data: 33.158983 N-79.211350 W Secondary Data: Wind direction, mean daily temperature, atmospheric conditions, and precipitation came from the Weather Underground Georgetown station [1]. Tidal and lunar data came from tides4fishing [2].
Data accessibility	With the article
Related research article	Wauson, M., Rogers, W. (2021) A test of the use of grey wolf (*Canis lupus*) urine to reduce coyote (*Canis lantrans*) depredation rates of loggerhead sea turtle (*Caretta caretta*) nests. DOI 126050. [3]

## Value of the Data

• The data show the number of loggerhead nests, as well as the number of those nests depredated by coyotes from 2015 to 2019 on South Island beach in the Tom Yawkey Wildlife Preserve, South Carolina. We evaluated the data in relation to the following abiotic factors: mean daily and nightly temperatures (C), tide type, atmospheric conditions, wind direction, lunar phase and nocturnal precipitation.
• Conservation groups can use the data to maximize the efficacy of night patrols, or other conservation efforts to reduce coyote depredation of loggerhead sea turtle nests.
• These data can be used when evaluating changes in behaviour regarding loggerhead nesting habits and possibly coyote depredation events. The data may also prove useful when recreating this experiment or in comparisons with the results of other conservation techniques.

## 1 Data Description

All the data below are from an Excel® spreadsheet that is part of the supplementary documents and is prepared so that it can be easily used in SPSS statistics software.

Table 1 shows the change in the distribution of coyote depredation events on loggerhead sea turtle nests as a function of the time interval between urine dispenser fillings. The data are only for the 2018 year. We broke the data into three categories; 9-day, 14-day, and 30-day. It took nine days for the urine in the dispensers to completely evaporate. Once this was discovered, we refilled the dispensers every 14-days for the second half of the experiment. Finally, the urine was advertised to be effective for 30-days, and so we include those data here as well.

**Table 1:** Change in depredation events as the length of time increases from when the dispensers were filled with urine.

	Treatment Area	Total-Control	Total
9-Days	1	13	14
14-Days	3	2	5
30-Days	7	6	13
Total	11	21	32

The data in this section are the total number of loggerhead nests made each year on South Island beach in relation to given abiotic factor. The totals vary as a result of incomplete historical data regarding that factor.

Table 2 shows the number of nests made each year with the corresponding atmospheric condition (whether the night was considered overcast or was clear for at least 51% of the night). A G-test demonstrated a significant depression on the number of nests made on clear nights compared with overcast nights (G-test adjusted with the Williams correction, G=13.197, df=1, p=0.00028).

**Table 2:** The number of loggerhead nests made each year when the skies where either clear or overcast for 51% of the night or more.

	Overcast	Clear	Total
2015	9	177	186
2016	56	185	241
2017	49	175	224
2018	61	101	162
2019	83	440	523
Total	258	1078	1336
Total Nights	97	317	414

Table 3 shows the number of nests made each year on South Island Beach and their relation to precipitation the nights those nests were made. A G-test displayed a significant reduction in the number of nests made on rainy nights compared with nights without precipitation (G-test adjusted with the Williams correction, G=14.155, df=1, p=0.000168).

**Table 3:** The number of loggerhead nests made each year on South Island Beach when there was no nocturnal precipitation compared to when there was.

	Precipitation	No Precipitation	Total
2015	26	164	190
2016	59	182	241
2017	76	150	226
2018	62	100	162
2019	91	432	523
Total	314	1028	1342
Total Nights	117	302	419

Table 4 shows the number of loggerhead nests made each year on South Island Beach based on the tide category present the night the nests were made. A G-test showed no statistical effect of nocturnal tide type with regards to loggerhead nest creation (G-test adjusted with the Williams correction, G=2.991, df=2, p=0.224).

**Table 4:** The number of loggerhead nests made each year on South Island Beach when there was only a high tide, low tide, or both tides between dusk and dawn*.*

	High Tide Only	Low Tide Only	Both Tides	Total
2015	58	42	90	190
2016	67	44	132	243
2017	49	39	138	226
2018	42	30	90	162
2019	126	106	291	523
Total	342	261	741	1344
Total Nights	108	74	238	420

Table 5 shows the number of loggerhead nests made each year on South Island Beach in relation to the predominant wind direction the night the nests were made. A G-test showed nocturnal wind direction had no significant effect on the number of nests made under any of the three conditions (G-test adjusted with the Williams correction, G=3.630, df=2, p=0.186).

**Table 5:** The number of loggerhead nests made each year on South Island Beach regarding the most prevalent wind direction for each night a nest was made.

	Landward	Seaward	Calm	Total
2015	112	25	53	190
2016	113	15	113	241
2017	84	12	130	226
2018	48	26	88	162
2019	95	133	295	523
Total	452	211	679	1342
Total Nights	158	59	202	418

Table 6 shows the number of nests made each year in relation to the possible lunar visibility on the beach. This does not take into account cloud cover, but simply measures the percent of light coming from the moon (new moon=0, full moon=100). A G-test demonstrated potential moon visibility had no significant effect on the number of nests made across the four categories (G-test adjusted with the Williams correction, G=1.197, df=3, p=0.754).

**Table 6:** The number of loggerhead nests made each year with relation to the percent lunar visibility (assuming cloudless night).

	0–24%	25–49%	50–74%	75–100%	Total
2015	50	38	32	70	190
2016	81	28	46	88	243
2017	70	37	57	62	226
2018	50	28	26	58	162
2019	154	85	83	201	523
Total	405	216	244	479	1344
Total Nights	130	69	72	149	420

Data in this section are the number of coyote depredation events on loggerhead nests the night they were laid as a function of the abiotic factors listed above. This section's nest totals are lower because they comprise only counts the nights when at least one loggerhead nests were made. The totals vary slightly as a result of incomplete historical data for the abiotic factor being evaluated.

Table 7 shows the number of coyote depredation events on loggerhead sea turtles the night the nest was made as a function of atmospheric conditions (whether the night was overcast or clear for at least 51% of the night). A G-test demonstrated atmospheric conditions had no significant effect on coyote depredation events (G-test adjusted with the Williams correction, G=2.316, df-1, p=0.128).

**Table 7:** The number of coyote depredation events on loggerhead sea turtle nests each year on South Island Beach in relation to the nocturnal atmospheric conditions.

	Overcast	Clear	Total
2015	3	65	68
2016	6	30	36
2017	12	42	54
2018	13	14	27
2019	16	76	92
Total	50	227	277
Total Nights	75	270	345

Table 8 shows the number of coyote depredation events on loggerhead sea turtle nests the night the nests were made and compares these events to the presence of nocturnal precipitation. A G-test showed no effect of nocturnal precipitation on the number of nests depredated (G-test adjusted with the Williams correction, G=1.151, df=1, p=0.283).

**Table 8:** The number of coyote depredation events on loggerhead sea turtle nests each year on South Island Beach in relation to the presence of any nocturnal precipitation.

	Precipitation	No Precipitation	Total
2015	10	59	69
2016	8	28	36
2017	17	37	54
2018	16	11	27
2019	17	75	92
Total	68	210	278
Total Nights	95	253	348

Table 9 shows the number of coyote depredation events on loggerhead sea turtle nests the night the nests were laid as a function of tidal category the night they were laid (high tide only, low tide only, both tides occurring). A G-test showed no effect of tide type on the number of nests depredated (G-test adjusted with the Williams correction, G=4.81, df=2, p=0.09)

**Table 9:** The number of coyote depredation events on loggerhead sea turtle nests each year on South Island Beach in relation to the type of tides present that night.

	High Tide Only	Low Tide Only	Both	Total
2015	22	22	25	69
2016	14	7	16	37
2017	11	10	33	54
2018	5	5	17	27
2019	29	13	50	92
Total	81	57	141	277
Total Nights	81	57	185	347

Table 10 shows the number of coyote depredation events on loggerhead sea turtle nests on the night the nests were laid as a function of the predominant wind direction for those nights (landward, seaward or calm). A G-test demonstrated no significant effect of wind direction on the number of depredation events (G-test adjusted with the Williams correction, G=0.427, df=2, p=0.808).

**Table 10:** The number of coyote depredation events on loggerhead sea turtles nests each year in relation to the primary wind direction for the nights the events took place.

	Landward	Seaward	Calm	Total
2015	6	47	16	69
2016	2	18	16	36
2017	4	23	27	54
2018	5	7	15	27
2019	21	16	55	92
Total	38	111	129	268
Total Nights	132	47	167	346

Table 11 shows the number of coyoted depredation events on loggerhead sea turtle nests on the night the nests were laid. It compares these events based on the potential lunar visibility on the beach. This does not take into account cloud cover, but simply measures the percent of light coming from the moon (new moon=0, full moon=100). A G-test demonstrated no significant effect of potential moon visibility on the number of nests depredated (G-test adjusted with the Williams correction, G=1.155, df=3, p=0.764).

**Table 11:** The number of coyote depredation events on loggerhead sea turtle nests each year in relation to the potential lunar visibility on the nights the events took place.

	0–24%	25–49%	50–74%	75–100%	Total
2015	20	14	15	20	69
2016	17	2	3	15	37
2017	19	7	12	16	54
2018	13	2	4	8	27
2019	24	17	17	24	92
Total	93	42	51	93	279
Total Nights	108	56	59	124	347

## 2 Experimental Design, Materials and Methods

South Carolina Department of Natural Resources Sea Turtle Technician teams collected nesting data every year from 17 May to 08 August from 2015 to 2019 on South Island beach at the Tom Yawkey Wildlife Center, S.C. Teams patrolled the beach each day at dawn; when they located a new nest, they recorded the turtle species based on the width of the carapace drag marks and the elbow joint impression in the sand. They also recorded the GPS coordinates and whether the nest was depredated. For depredated nests, the technicians identified the predator by the tracks it left in the sand.

During the 2018 data collection period, we placed urine dispensers four meters apart along 4100 meters along the first dune line parallel to the ocean on South Island Beach. This area consisted of three types of zones. The zones alternated from Treatment to Partial Control to Control back to Partial control and repeated that pattern for a total of 27 zones starting with a Treatment region and ending with a Control (Supplementary figure). Each treatment and control zone was 200 meters, while the partial control zones were only 100 meters. We combined the partial-control and control regions into a single entity, ``Total Control'', because we later established the two were statistically indistinguishable At the end of each treatment and control zone, the dispensers formed a perpendicular transect from the first dune line to 4 meters above the king tide line.

Every zone had urine dispensers, but only the treatment zone had wolf urine in the dispensers. Following the manufacturer's guidelines (Maine Outdoor Solutions), we placed the dispensers four meters apart down the beach's length. Each dispenser consisted of a 25.4 cm wooden post with a small hole on one end, a cap, a vial with holes near the top across from each other holding 44.36 mL of urine, and a plastic tie to secure all of the components.

We collected environmental data to determine whether there were any correlations with when a nest was more likely to be made or depredated. That data included high and low tide levels (m), mean daily temperatures (C), mean nightly temperatures (C), moon phase groups (lunar visibility groupings), nocturnal atmospheric conditions (clear sky, overcast), whether there was nocturnal rain, and nocturnal wind direction (landwards, seaward, or calm). Wind direction, mean daily temperature, mean nightly temperature, atmospheric conditions, and precipitation data came from Weather Underground [1]; we obtained tidal and lunar data from Tides4Fishing [2]. South Carolina Department of Natural Resources provided the nesting and depredation data. We evaluated each environmental factor's effect across all five years but excluded the depredation data in the treatment zones for 2018.

### 2.1 Statistical tests

To evaluate the effect of wolf urine on coyote depredation of loggerhead sea turtle nests, the data were broken into two groups, one based on the time it took the urine to evaporate, 9-days, and the other for the length of time the manufacturer stated the wolf urine would be active, 30-days. We initially analyzed the partial-control areas as a separate date zone from the treatment (contained urine) and control (no urine). However, the partial-control was statistically indistinguishable from the control areas in all aspects, and so was combined with the control to form the “total-control” group. This total-control group occupied 2700 meters of the 4100-meter experimental area. We used the G-test with the Williams correction to compare the frequencies of depredated and non-depredated nests in the treatment vs. total-control areas.

We used G-tests to examine the effects of atmospheric conditions, nightly precipitation, wind direction, and moon phase groups on the number of loggerhead nests made and the number of loggerhead nests depredated by coyotes each night. If we found an effect, we used Z-scores to determine the influential cells. We used Spearman's rho to test whether a correlation existed between mean daily or nightly temperatures and the number of nests laid or depredated per night.

## Ethics Statement

Besides part of the data collection for 2016 when one of the authors was a member of the South Carolina Department of Natural Resource Turtle Technician Team, neither of the authors saw or touched a sea turtle, egg, or coyote throughout the experiment. We only handled the urine dispensers on the beach. All nests and eggs were handled only by the trained SCDNR Sea Turtle Technician Team. As a result of this design, the experiment was approved by the Institutional Animal Care and Use Committee at Winthrop University (IACUC Approval # 18004). No DNR permit was required or requested for the completion of this research.

## CRediT Author Statement

**Michael Wauson**: Conceptualization, Methodology, writing- Original Draft preparation, Visualization, Investigation, Data Curation; **William Rogers**: Writing-Reviewing and Editing, Supervision, Formal Analysis, Validation, Methodology, Funding Acquisition, Resources

## Declaration of Competing Interest

The authors declare that they have no known competing financial interests or personal relationships which have or could be perceived to have influenced the work reported in this article.

## Appendix Supplementary materials

Image, application 1
